# Community Knowledge, Attitudes and Preventive Behaviour Towards the Cardiovascular Benefits of Reduced Exposure to Air Pollution in Nigeria: Evidence from the CARDINAL Study

**DOI:** 10.5334/gh.1482

**Published:** 2025-10-09

**Authors:** Adekunle Gregory Fakunle, Temilade Bello, Akintayo Olamide Ogunwale, Oyewale Mayowa Morakinyo, Olubunmi Ayinde, Susan Motunrayo Kebu, Oluwapelumi Peter Arinola, Marvelous Adeoye, Bosede Adebayo, Iretioluwa Mary Bamtefa, Akinkunmi Paul Okekunle, Augustine Odili, Mark R. Miller, Amam C. Mbakwem, Abiodun Moshood Adeoye

**Affiliations:** 1 Department of Public Health, College of Health Sciences, Osun State University, Osogbo, Osun State, NG; 2 Department of Occupational and Environmental Health, School of Nursing and Public Health, University of KwaZulu-Natal, Durban, ZA; 3 Department of Public Health, College of Health Sciences, Bowen University, Iwo, Osun State, NG; 4 Department of Environmental Health Sciences, Faculty of Public Health, University of Ibadan, NG; 5 Department of Epidemiology and Medical Statistics, Faculty of Public Health, University of Ibadan, Ibadan, NG; 6 Department of Medicine, Cardiology Unit, College of Medicine, University of Ibadan, NG; 7 Institute of Public Health and Wellbeing, University of Essex, Colchester, UK; 8 Department of Paediatrics, University of Ibadan, Ibadan, NG; 9 Department of Nutrition, Seoul National University, 08826, KR; 10 Department of Medicine, University of Abuja, FCT, NG; 11 Centre for Cardiovascular Science, The University of Edinburgh, Queens Medical Research Institute, Edinburgh, UK; 12 Department of Medicine, College of Medicine, University of Lagos, NG

**Keywords:** knowledge, attitude, behaviour, air pollution, cardiovascular diseases, Nigeria

## Abstract

**Background:**

Air pollution has emerged as a known risk factor for cardiovascular diseases (CVDs) globally. Raising public knowledge of the importance of air pollution exposure is crucial for implementing future interventions to improve cardiovascular health. This study aimed to explore the knowledge, attitude and behaviour (KAB) of vulnerable women and men towards the cardiovascular benefits of reducing air pollution exposure.

**Methods:**

A cross-sectional study was conducted among 602 vulnerable men and women in Ibadan, Nigeria, using a multi-stage sampling technique. Using the KAB framework, emphasis was placed on the link between air pollution exposure reduction and the burden of CVDs such as stroke, heart failure, heart attack, congenital heart disease, cardiac arrest and atherosclerosis. Data were collected using a digitalised validated semi-structured questionnaire that included questions on knowledge of the link between air pollution and CVD, attitude towards reducing air pollution and behaviours related to reducing air pollution exposure. The median (interquartile range [IQR]) KAB scores were calculated and dichotomised using the median score. Data were analysed using descriptive statistics, chi-square, Spearman’s correlation analysis and regression models at *p* < 0.05.

**Results:**

Respondents’ mean age was 44.1 ± 14.0 years, and 54.2% were females. The participants’ median (IQR) knowledge score was 7.0 (2.0–8.0), with the majority, 66.9%, having poor knowledge. The median (IQR) pollution-reduction attitude score was 10.0 (3.0–16.0), with a majority (58.5%) having a negative attitude. Respondents’ median preventive behaviour score was 6.0 (1.0–11.0), and 58.6% had unsatisfactory behaviour. Awareness about air pollution was found to be associated with knowledge (aOR [adjusted odds ratio] 0.82; 95%CI: 0.57–0.97) and behaviour (aOR 0.44; 95%CI: 0.31–0.64) towards air pollution reduction.

**Conclusion:**

The poor knowledge of the link between exposure to air pollution and CVD underscores the need for targeted educational initiatives, supported by regulatory interventions, to harness the cardiovascular health benefits of reduced exposure to air pollution in Africa.

## Introduction

Air pollution remains the most significant environmental risk factor for human health globally (1). Over half of the world’s population resides in cities due to rapid population transition and urbanisation, and by 2050, the number is expected to rise to 68% (2). Although one of the most important principles of preserving health and well-being is maintaining clean air, this principle is often overlooked (3,4). Air pollution levels have increased in major urban centres primarily due to factors such as high vehicular emissions, industrial activities and burning of fossil fuels, especially in developing countries. According to a report by the World Health Organization (WHO), 90% of the global population resides in regions where pollutants exceed air quality guidelines needed to protect health (5). Around 78% of carbon emissions and significant airborne pollutants are produced in urban cities and negatively impact more than 50% of the world’s population (6). In addition, WHO in 2019 estimated that ambient (outdoor) air pollution was responsible for 4.2 million premature deaths globally, and nearly 90% of these deaths occurred in low- and middle-income countries (LMICs), such as Nigeria (5,7).

Indoor burning of solid fuels is the dominant source of household air pollution (HAP), especially in developing countries (8). When burnt, solid fuels produce several pollutants, including particulate matter and gases of sulfur, nitrogen and carbon that affect human health and cause several health burdens, including cardiovascular diseases (CVDs) (9). In developing countries such as Nigeria, women disproportionately suffer from the impact of this burning, given that they are constantly responsible for cooking, accounting for 5% of all female deaths (10). This confines women to a particularly higher risk owing to the prolonged duration of time spent in the kitchen compared to men (11). On the other hand, ambient air pollution significantly impacts individuals who spend most of their time outdoors (12), including the commercial motor drivers of buses, cars and motorcycles. These occupations are mainly held by men, representing an important part of the labour force, especially in LMICs (13). The vehicles they drive are both sources of air pollution for the drivers and others who work in the outdoor environment. Considering the level of exposure of these vulnerable populations of women and men, their knowledge, attitude and preventive behaviour (KAB) towards air pollution reduction have not been extensively explored, especially in developing countries.

The significant health effects of exposure to air pollution are now well established, with a growing recognition that air pollution has major adverse effects on all organs of the human body (14). Long-term or short-term exposure to air pollutants increases the risk of several health conditions, including cancer (15,16), respiratory (17,18) and cardiovascular (19,20) diseases, leading to elevated morbidity and mortality. In 2016, WHO reported that almost 58% of ambient air pollution-related premature deaths were due to ischaemic heart disease (IHD) and stroke, while 18% of deaths were due to chronic obstructive pulmonary disease (COPD) and acute lower respiratory, and 6% of deaths were due to lung cancer (21). Chronic exposure to air pollution has been linked to a 5–10% increased risk of myocardial infarction, stroke, heart failure and sudden death onset (22). The risk increases by 1–2% for every 10 µg/m3 increase in particulate matter size 2.5 microns (PM_2.5_) concentrations (23).

In Nigeria, unregulated emissions from power plants, malfunctioning transportation systems and the increased rate of industrial and construction activities have contributed immensely to the increasing levels of air pollution. The uncontrolled emissions from gas flaring and other petroleum refining activities are also major causes of air pollution concerns in the country (24,25). In 2015, over 51% of the total gases produced in Nigeria were reported flared, accounting for between 10,500 and 5,100,00 tons of pollutants emitted, including PM (26). According to a report by the Nigeria Bureau Statistics, the total number of registered cars on Nigerian roads increased to about 11.8 million in the year 2018, with almost 630,868 additional driver’s licenses issued that same year (27). These vehicles run on fossil fuels, which account for over 90% of the total consumption of petroleum products in Nigeria (28). Government-level solutions to reduce the level of exposure to air pollution depend on industrial transformation and require a cumulative effort before the desired resultant effect can be observed. These solutions include enforcing regular servicing of commercial motor vehicles, reducing the use of unclean fuel and achieving emission-reduction targets which could all yield long-term benefits. In addition, designing interventions targeted at improving knowledge about air pollution might be promising in promoting lifestyle approaches for preventing or reducing air pollution exposure, thereby improving cardiovascular health. However, data on this phenomenon are scarce, especially among vulnerable populations from LMICs.

The KAB framework explains that a person’s knowledge directly influences their attitudes, which in turn, indirectly affects their healthcare-related behaviours and decision-making (29). According to this framework, it can be assumed that individuals’ knowledge about air pollution and CVDs shapes their attitudes towards these issues, and that their attitudes, in turn, influence the actions (behaviours) they take. Given the potential long-term effect of air pollution on quality of life, there is a paucity of data on the knowledge of community members regarding the cardiovascular benefits of reduced exposure to air pollution. This study aims to gather baseline data on the knowledge gap regarding air pollution and cardiovascular health to inform a more extensive intervention study. The CARDINAL study was comprehensively designed to evaluate the impact of a novel AirHealth educational intervention on community members’ KAB towards indoor and outdoor air pollution reduction strategies. Through this survey, we seek to assess pertinent antecedent factors associated with the knowledge, attitude and perception of community members on the cardiovascular benefits of reduced exposure to air pollution, with the ultimate goal of informing strategies and policies to address this critical environmental issue in Nigeria.

## Methodology

### Study design, population and eligibility

This baseline assessment is part of the Community-Based Cardiovascular Risk Reduction through Novel Air Health Behavioural Change Intervention (CARDINAL) project, which is a pre- and post-interventional community-wide assessment to evaluate the impact of a novel AirHealth intervention. Details on the CARDINAL protocol have been published (30). Briefly, the study was conducted in Ibadan North Local Government Area (LGA) of Ibadan, Oyo State, Nigeria, which is primarily inhabited by Yoruba speakers. The population consists mainly of traders, artisans, civil servants and students. The study included vulnerable populations of men (commercial transport workers) and women (exposed to indoor burning of solid fuel) who were ≥18 years old and had been residing in the selected communities for more than 12 months.

### Sample size determination, community entry and sampling strategy

The sample size calculation for the CARDINAL study has been published elsewhere (30). Briefly, assuming an absolute proportional change in a specific KAB component of 0.13 with a standard deviation of 2.5 at 80% power and a drop rate of 10%, a minimum size of 300 participants in each of the vulnerable populations of men and women will be sufficient to observe a difference of approximately 2.1. In addition, details of the CARDINAL community entry through the ward development committee (WDC) have also been previously described (30). A total of five distinguished persons from the different wards in Ibadan North LGA were identified and recruited to form the Community Advisory Network (CAN). In total, 300 commercial transport workers and 300 reproductive-aged women were identified and recruited using a multi-stage sampling technique, as described in the protocol (30). In stage one, a purposive selection of five wards from a total of 12 wards in Ibadan North LGA where the target population of vulnerable men and women resides, was carried out. This was followed by a clustering of communities based on shared characteristics such as the presence of associations of women, caterers, charcoal sellers, firewood sellers and commercial transport (tricycle, motorcycle and bus) drivers/workers. Therefore, two clusters were selected from each ward using simple random sampling with the aid of balloting procedure. Subsequently, site visits were carried out, which allowed us to purposively select one community per cluster, prioritising those with the highest population size and density. In stage three, eligible groups of women, including caterers, charcoal sellers, firewood sellers and men who were commercial transport (tricycle, motorcycle and bus) workers were identified in each selected cluster. After this, a simple random sampling using balloting of women from women’s associations (50% from general women group, 20% from caterers’ association, 20% from charcoal sellers’ group and 10% from firewood sellers’ group) and men from the commercial transport workers’ associations (50% from bus, 30% motorcycle and 20% tricycle drivers) was carried out in stage four until the required sample size was obtained.

### Data collection method and tools

The instrument for data collection was a pre-tested semi-structured AirHealth questionnaire, which had a Yoruba language translation to ensure clarity and detailed understanding of the questions. The tool contained inquiries on awareness about air pollution, knowledge of air pollution reduction strategies, knowledge of CVDs and the link with air pollution, attitude towards air pollution reduction and perception relating to air pollution reduction. The questions were developed through a review and adaptation of items from previous studies (31,32,33,34,35,36) by a multidisciplinary team of trained experts. The instrument underwent validation by a group of public health experts and was pretested in another community that was not part of the main study before its finalisation. The questionnaire consisted of eight sections. The first section asked about the sociodemographic characteristics of the participant, including age assessed quantitatively, religion categorised as christians, muslims and traditionalists, the highest level of education assessed as ‘no formal education’, ‘primary’, ‘secondary’ and ‘tertiary’, respondents’ income collected in dollars and so on. The second and third sections consisted of a 11-point and 10-point scale on air pollution awareness and knowledge of air pollution reduction strategies. In the fourth section, an 8-point scale was used to assess the knowledge of community members on the link between air pollution and CVDs. The fifth section contained a 16-point Likert scale with five levels on the attitude of participants towards air pollution reduction strategies, ranging from strongly agree to strongly disagree, while the sixth section assessed the behaviour of participants towards air pollution exposure reduction using a 12-point behavioural scale. After validation, the questionnaire was digitalised using the KoBo collect toolbox for easy administration (37). The general meeting periods of the women’s and men’s associations in the selected communities were targeted for data collection by the research assistants (RAs). Data collected were stored in the cloud and later transferred to SPSS for analysis. Data were collected with the help of 12 trained RAs. The RAs were individuals with at least a master’s degree in any science/health discipline, capable of speaking Yoruba and a good understanding of Ibadan North LGA terrain. The RAs were exposed to a 2-day training covering the aims and objectives of the CARDINAL project with specific emphasis on the methodology, the definition of essential variables and the instrument for data collection (AirHealth questionnaire) and ethical issues involved in the study. At the end of every recruitment, the RAs met with the study team weekly for debrief.

The level of knowledge on the link between exposure to air pollution and CVD was assessed using a 10-point knowledge scale. A correct answer for each item was scored ‘1’ and an incorrect answer was scored ‘0’. Using the median knowledge score, ≤6.0 was categorised as poor knowledge, while >7.0 was considered as good knowledge. Attitude towards air pollution reduction strategies was assessed using a 16-point attitude scale. Depending on the direction of the question on the Likert scale, strongly agree was coded ‘5’ while strongly disagree was coded ‘0’. A score above the median attitude score, >10.0, was categorised as a positive attitude, while ≤10.0 was categorised as a negative attitude. Furthermore, preventive behaviour towards air pollution reduction was assessed using an 11-point scale. A correct answer for each item was scored ‘1’ and an incorrect answer was scored ‘0’. Using the median behaviour score, >6.0 was considered as satisfactory behaviour, while ≤6.0 was categorised as unsatisfactory behaviour. In this study, KAB scores were dichotomised in line with several KAB studies (38,39,40).

### Data processing and analysis

Demographic characteristics of the participants were described using absolute and relative frequencies. Continuous data were presented as median (interquartile range [IQR]), while categorical data were presented as frequencies and proportions. The KAB scores were presented with a box plot due to the lack of compliance with the normality assumption. The bivariate analyses between sociodemographic characteristics and KAB categories were conducted using cross tabulation, whereas the quantitative variables were compared using Mann–Whitney *U*. Finally, multi-variable regression models were performed to identify sociodemographic and lifestyle factors associated with KAB. All statistical analyses were conducted using SPSS version 27 and R programme version 4.0. Data were analysed at 95% level of significance.

## Results

### Sociodemographic characteristics of the participants

The study included 602 respondents, with a mean age of 44.1 ± 17.0 years. Respondents <50 years of age constituted 62.1%. Females were slightly more represented, accounting for 54.2% of participants. The dominant religion was Islam (69.0%), followed by Christianity (30.8%). Most (87.6%) of the respondents were from the Yoruba ethnic group. The majority (73.6%) of the respondents were from monogamous families, and almost half (49.6%) had secondary education as their highest level of education (Table 1).

**Table 1:** Sociodemographic characteristics of the respondents.


SOCIODEMOGRAPHIC CHARACTERISTICS	FREQUENCY (*n* = 607)	PERCENTAGE (%)

Age (years)		

**Mean ± SD**	**44.08 ± 17.03**	5.4

<20	33	32.3

20–29	196	0.0

30–39	0	20.1

40–49	122	19.4

50–59	118	12.9

60–69	78	6.8

70–79	41	3.1

≥80	19	

Age (years)		

<50	377	62.1

≥50	230	37.9

Sex		

Male	278	45.8

Female	329	54.2

Religion		

Christianity	187	30.8

Islam	419	69.0

Traditional	1	0.2

Ethnicity		

Yoruba	532	87.6

Igbo	15	2.5

Hausa	60	9.9

Marital Status		

Single	116	19.1

Married	420	69.2

Separated	5	0.8

Divorced	8	1.3

Widowed	58	9.6

Type of family		

Monogamy	447	73.6

Polygamy	160	26.4

Highest level of education		

No formal education	86	14.2

Primary	158	26.0

Secondary	301	49.6

Tertiary	62	10.2

Household size		

<5	271	44.7

≥5	336	55.4

Respondent’s income		

<$100	559	92.1

≥$100	48	7.9

Spouse’s income		

<$100	584	96.2

≥ $100	23	3.8

Length of stay in years		

<1	17	2.8

1–3	100	16.5

4–6	80	13.2

≥7	410	67.5


### Awareness and knowledge of the link between air pollution and CVD

**Figure 1:**
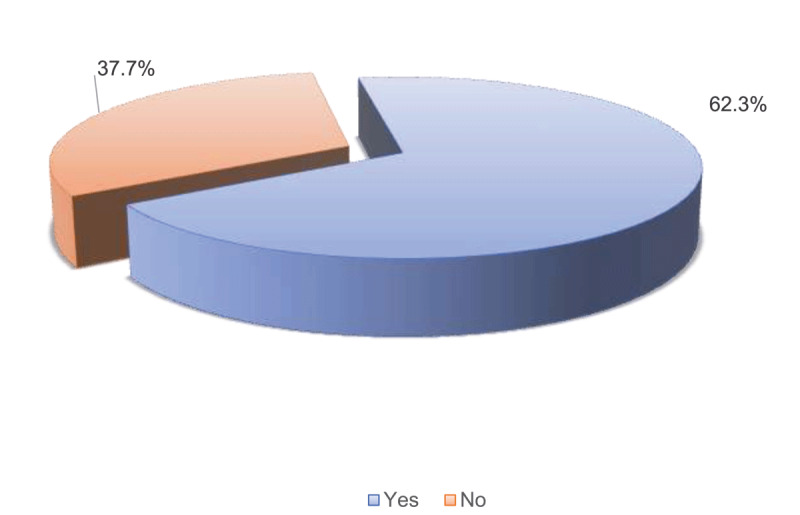
Air pollution awareness.

**Figure 2:**
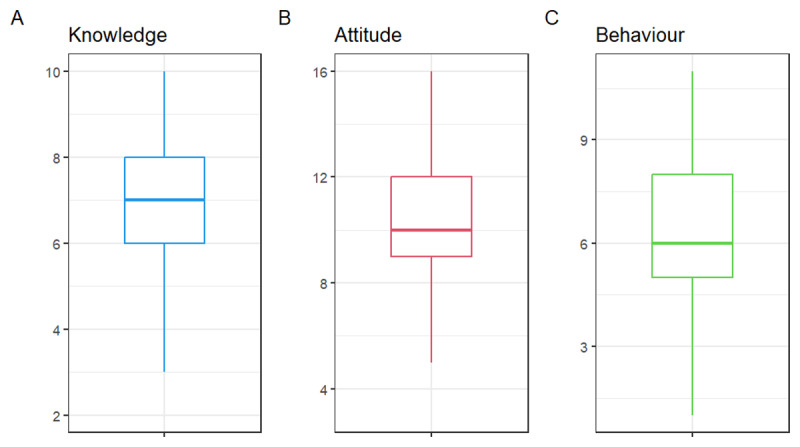
A box plot representing the median knowledge **(A)**, attitude **(B)** and behaviour score **(C)**.

**Figure 3:**
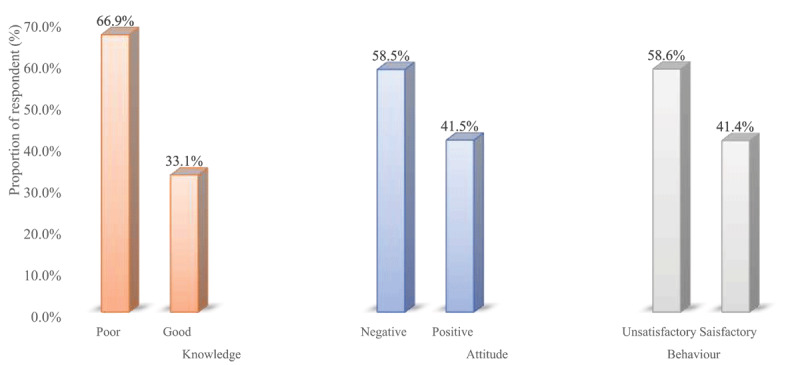
Proportion of respondents with poor vs. good knowledge; negative vs. positive attitude; and unsatisfactory vs. satisfactory behaviour.

Regarding awareness about air pollution, the majority, 62.3% of respondents, were found to be aware of air pollution (Figure 1). As many as 68.0% identified air pollution as a contributor to stroke, and 65.7% acknowledged its potential to exacerbate chronic heart failure. In addition, 55.7% noted its role in sudden cardiac death, while 51.6% indicated that air pollutants could result in a heart attack (Table 2). In addition, participants demonstrated varied knowledge of air pollution reduction measures. 85.0% reported that biogas usage for cooking could reduce pollution, and 83.4% mentioned the importance of regular motor engine servicing. However, less than half (46.8%) supported public transportation usage as a strategy, and 49.4% suggested using charcoal instead of firewood for cooking. In addition, a little above half (50.7%) of respondents supported biking as a strategy for reducing air pollution (Table 3). The overall median (IQR) knowledge score on the link between air pollution and CVD was 7.0 (IQR: 2.0–10.0), with the majority (66.9%) being classed as having poor knowledge of the link between air pollution and CVD (Figures 2 and 3).

**Table 2:** Knowledge of respondents on the link between air pollution and CVD.


AIR POLLUTION CAN LEAD TO:	FREQUENCY (*n* = 607)	PERCENTAGE (%)

**Lead to stroke**		

No	194	32.0

Yes	413	68.0

**Lead to an increase in blood pressure**		

No	267	43.9

Yes	340	56.1

**Can enter the bloodstream**		

No	338	55.7

Yes	269	44.3

**Enter lung only without causing CVD**		

No	252	41.5

Yes	355	58.5

**Result in heart attack**		

No	294	48.4

Yes	313	51.6

**Exacerbate chronic heart failure**		

No	288	44.3

Yes	399	65.7

**Lead to sudden cardiac death**		

No	269	44.3

Yes	338	55.7

**Reduce blood pressure**		

No	267	44.0

Yes	339	56.0


CVD: cardiovascular disease.

**Table 3:** Knowledge of respondents on air pollution reduction strategies.


WHICH OF THE FOLLOWING DO YOU THINK CAN REDUCE AIR POLLUTION	FREQUENCY (*n* = 607)	PERCENTAGE (%)

Use of public transportation		

No	323	53.2

Yes	284	46.8

Use of charcoal instead of firewood for cooking		

No	307	50.6

Yes	300	49.4

Tree planting		

No	276	45.5

Yes	331	54.5

Use of air conditioning system at home		

No	202	33.3

Yes	405	66.7

Use of biogas for cooking		

No	91	15.0

Yes	516	85.0

Use of electric stove		

No	104	17.1

Yes	503	82.9

Open burning in the neighbourhood		

No	218	35.9

Yes	389	64.1

Opening of windows while cooking indoors		

No	125	20.6

Yes	482	79.4

Encourage biking		

No	309	50.7

Yes	299	49.3

Regular servicing of motor engines		

No	101	16.6

Yes	506	83.4


### Attitude towards air pollution reduction strategies

Table 4 presents the issues used in the assessment of the attitude of respondents towards air pollution reduction strategies. Most (84.5%) respondents agreed that community members have a role in reducing air pollution. Strong enforcement of environmental laws was considered as an effective solution by 85.2% of respondents, while 83.9% agreed that banning open burning and dumping could contribute to reducing air pollution. However, 33.4% incorrectly agreed that using firewood for cooking does not impact air pollution. In addition, almost half (49.6%) of the respondents disagreed with cutting down trees as a strategy for reducing air pollution. The attitude ranged from 3.0 to 16.0 with a median score of 10.0. More than half (58.5%) of the respondents had an overall negative attitude towards air pollution reduction strategies.

**Table 4:** Attitude of respondents towards air pollution reduction strategies.


	FREQUENCY (*n* = 607)	PERCENTAGE (%)

All community members have a role to play in reducing air pollution		

Disagree	80	13.2

Undecided	14	2.3

Agree	513	84.5

Government banning the use of petrol cars can help reduce air pollution		

Disagree	189	31.1

Undecided	50	8.2

Agree	368	60.6

The strong enforcement of environmental protection laws that prohibit air pollution is a good way to start preventing it		

Disagree	65	10.7

Undecided	25	4.1

Agree	517	85.2

Air pollution reduction is strictly Government affair		

Disagree	404	66.6

Undecided	45	7.4

Agree	158	26.0

Open burning and open dumping should be banned to control air pollution		

Disagree	67	11.0

Undecided	31	5.1

Agree	509	83.9

The use of firewood for cooking is an ancient cooking method that doesn’t have anything to do with air pollution		

Disagree	355	33.4

Undecided	49	8.1

Agree	203	33.4

Cutting down trees is a good way to reduce air pollution		

Disagree	301	49.6

Undecided	63	10.4

Agree	243	40.0

Periodic servicing of automobile engine has nothing to do with air pollution reduction		

Disagree	143	73.0

Undecided	21	3.5

Agree	443	23.6


### Behaviour of respondents towards air pollution reduction

Issues relating to the behaviour of respondents towards air pollution reduction are shown in Table S1. Most (91.2%) of respondents were willing to reduce air pollution exposure to improve their health, and 91.9% indicated readiness to shift from solid fuels to biogas for cooking if available. However, only 22.6% had received advice on air pollution from social or governmental sources, and less than half (43.9%) of the respondents had ever used real-time air quality information to guide their activities (Table 5). The overall median (range) behaviour score was 6.0 (IQR: 1.0–11.0), with majority, 58.6% categorised as having unsatisfactory behaviour.

**Table 5:** Behaviour of respondents relating to air pollution reduction.


BEHAVIOUR	FREQUENCY(*n* = 607)	PERCENTAGE (%)

Do you know how to perform a seal check on facemask?		

No	379	62.4

Yes	228	37.6

Do you find the use of face mask comfortable during exposure to air pollution?		

No	158	26.0

Yes	449	74.0

Are you ready/willing to substitute biomass fuel (wood, sawdust, kerosene stove) with cleaner fuels such as gas, electric or solar cooker?		

No	45	7.4

Yes	562	92.6

Have you received any form of education on the health risks associated with burning solid fuels for cooking and heating?		

No	404	66.6

Yes	203	33.4

Would you rather consider the use of active transport system such as walking or cycling in a bid to reduce air pollution exposure?		

No	318	52.4

Yes	289	47.6

Can your financial capacity allow you to switch from biomass fuel to cleaner fuels?		

No	251	41.4

Yes	356	58.6

Have you considered the use of real-time information on local air quality from sources such as mobile phone applications, news feeds and websites, to guide route and timing for your movement?		

No	341	56.1

Yes	266	43.9

Have you received any form of advice from your local health care workers on local air quality monitoring so as to minimize exposure?		

No	387	63.8

Yes	220	36.2

Do you have any social group/government source educating you on how to reduce exposure to air pollution in your community?		

No	470	77.4

Yes	137	22.6

Will you be willing to reduce exposure to air pollution to improve your health status?		

No	54	8.9

Yes	553	91.2

If a cleaner stove is made available, will you be willing to shift from solid fuel to biogas for cooking?		

No	49	8.1

Yes	558	91.9


### Correlation and sociodemographic factors associated with KAB categories

The bivariate analysis revealed that age (*p* = 0.035), ethnicity (*p* = 0.013), religion (*p* = 0.033), education (*p* = 0.001) and family type (*p* = 0.01) were influencing factors for knowledge of the link between air pollution and CVD. Attitude towards air pollution reduction strategies was found to be associated with ethnicity (*p* = 0.021), family type (*p* = 0.030) and household size (*p* = 0.035). Factors associated with preventive behaviour towards air pollution reduction include sex (*p* = 0.001), education (*p* < 0.001), and awareness about air pollution (*p* < 0.001) (Table S1). A weak but statistically significant correlation was observed between knowledge and attitude (*r* = 0.32, *p* < 0.001), and between knowledge and behaviour (*r* = 0.13, *p* = 0.001) (Table S2). In the multivariate regression model, respondents who were aware of air pollution were 18.0% and 56.0% less likely to have poor knowledge (aOR 0.82, 95%CI: 0.57–0.97) and unsatisfactory behaviour (aOR 0.44, 95%CI: 0.31–0.64), respectively, as compared to those who were unaware. Also, males were 42.0% less likely to exhibit unsatisfactory behaviour (aOR 0.58, 95%CI: 0.41–0.83) as compared to females. In addition, monogamy was an independent predictor of poor knowledge (aOR 1.41, 95%CI: 1.03–2.12) and negative attitudes (aOR 1.54, 95%CI: 1.04–2.30) (Table 6 and Figure S1).

**Table 6:** Adjusted multivariate analysis of sociodemographic factors associated with KAB.


PREDICTORS	POOR KNOWLEDGE; AOR (95% CI)	NEGATIVE ATTITUDE; AOR (95% CI)	UNSATISFACTORY BEHAVIOUR; AOR (95% CI)

**Awareness about air pollution**			

No	1.00	1.00	1.00

Yes	**0.82 (0.57–0.97)**	0.99 (0.70–1.42)	**0.44 (0.31–0.64)**

**Age (years)**			

≤50	1.00	1.00	1.00

>50	0.83 (0.55–1.25)	0.93 (0.63–1.38)	0.96 (0.64–1.44)

**Sex**			

Male	0.94 (0.65–1.36)	0.75 (0.53–1.06)	**0.58 (0.41–0.83)**

Female	1.00	1.00	1.00

**Ethnicity**			

Yoruba	0.17 (0.01–1.33)	**0.10 (0.01–0.75)**	0.91 (0.31–2.71)

Hausa	0.09 (0.01–1.02)	**0.11 (0.01–0.89)**	1.16 (0.35–3.86)

Igbo	1.00	1.00	1.00

**Type of family**			

Polygamy	**1.41 (1.03–2.12)**	**1.54 (1.04–2.30)**	1.03 (0.68–1.57)

Monogamy	1.00	1.00	1.00

**Highest level of education**			

No formal education	1.00	1.00	1.00

Primary	0.74 (0.42–1.30)	0.62 (0.35–1.10)	0.76 (0.49–1.19)

Secondary	1.36 (0.77–2.41)	0.74 (0.42–1.31)	0.57 (0.32–1.04)

Tertiary	**0.67 (0.04–0.92)**	0.99 (0.48–2.15)	**0.42 (0.20–0.89)**

**Household size**			

<5	1.00	1.00	1.00

≥5	1.23 (0.85–1.78)	**1.49 (1.05–2.11)**	0.85 (0.60–1.21)


KAB: knowledge, attitude and behaviour; CI: confidence interval; aOR: adjusted odds ratio.

## Discussion

Air pollution remains one of the poorly studied environmental stressors for health in Africa (41). Awareness of the cardiovascular effects of air pollution may be especially poor, despite the increasing scientific evidence showing that air pollution is linked to high levels of cardiovascular morbidity and mortality. To the best of our knowledge, this is the first study in Nigeria to evaluate the KAB of community members regarding the cardiovascular benefits of reduced exposure to air pollution. Identifying the limitations in awareness in this area could help guide the implementation of air quality strategies and improve the effectiveness of interventions addressing environmental health risk factors in African communities.

Our study found an overall poor knowledge of the link between air pollution and CVD among the majority of the community members. A notable contributor was the differences in knowledge among more than half of the participants on the possibility that air pollutants can pass into the bloodstream, to promote oxidative stress, cardiovascular effects and embolism (42). Therefore, improving the knowledge of community members on the cardiovascular impact of exposure to air pollution may be an essential tool for behavioural modification and possible improvement in cardiovascular health. In addition, the poor knowledge of the link between air pollution and CVD was associated with air pollution awareness and the educational status of the respondents. Participants who were aware of air pollution were less likely to have poor knowledge of the link with CVD. Our report resonates with the fact that education is the primary means of acquiring and accumulating knowledge (43). This necessitates the need for awareness creation and improved education on air pollution and underscores the need for targeted educational campaigns to emphasise the cardiovascular risks associated with air pollution. Appreciation of the lifelong burden of CVD due to early and sustained exposure to pollutants (44) may help reinforce awareness building.

Another major finding is the negative attitude towards the air pollution reduction strategy recorded among respondents. Most of the respondents reported that community participation is critical to reducing air pollution, and 85.2% advocated for a strong enforcement of environmental laws. These attitudes resonate with those raised by Ramírez et al. (45), who emphasised the importance of public engagement and policy enforcement in environmental health literacy campaigns. Interestingly, some respondents erroneously believed that using firewood for cooking does not contribute to air pollution. Agbo et al. (46) documented the significant role of biomass combustion in air pollution in the African context. The negative attitude towards air pollution reduction strategies was associated with family type and increased household size. As evident in the current study, individuals belonging to a polygamous family are approximately two times more likely to display a negative attitude towards available air pollution reduction strategies. According to Marín et al. (47), an overpopulated environment, such as a polygamous household, has the potential to trigger negative perceptions and less willingness to act (47), probably due to direct or indirect influence of members of the household. It is important to note that good knowledge can foster a positive attitude (48), although both entities are distinct. For example, a considerable proportion of respondents disagreed that using firewood for cooking does not impact air pollution. This positive attitude might be the result of good knowledge about the contribution of solid fuel use to indoor air pollution. This claim was further supported by the direct positive correlation recorded between knowledge and attitude in the current study. A positive attitude can encourage learning and the application of knowledge, while poor knowledge can lead to a negative reaction (49). A positive behavioural intention was observed among respondents, with almost all respondents willing to reduce air pollution exposure and ready to switch from solid fuels to biogas if available. Marcantonio et al. (50) emphasise the role of access to clean energy sources in fostering environmentally conscious behaviours. However, only 23% had received advice on air pollution from social or governmental sources, suggesting a significant gap in public health communication. The observed economic constraint in terms of respondents’ income could impact individuals’ ability and willingness to change their behaviour (51) towards reducing exposure to air pollution. The likelihood of exposure to HAP has been associated with low household income (52,53). For example, the inability to afford cleaner fuel could prevent its usage despite the willingness to substitute solid fuel use with cleaner fuel. Therefore, due to the financial limitations experienced by the majority of the population studied, they tend to prioritise immediate needs such as food and shelter over long-term environmental conscious behaviour, leading to less investment in air pollution-reduction strategies. The usage of real-time air quality information by only 44% of respondents highlights the need for increased availability and dissemination of such data, as advocated by Yu et al. (54). Sex disparities were evident, with males more likely to exhibit satisfactory behaviour compared to females, who demonstrated poorer behavioural outcomes. The observed disparity in the sex-specific behaviour could be due to the diverse usage of fuel within and between the populations. This expected diversity in the mode and pattern of fuel usage across different populations is likely to influence their preventive behaviour to make the necessary changes. This aligns with Isezuo et al. (55), who suggest the need for sex-specific interventions to address behavioural gaps. Although weak, the direct correlation between knowledge and attitude supports the theory that informed individuals are more likely to develop favourable attitudes towards pollution reduction (56). However, attitude in the current study did not translate into behavioural modification, probably due to the role of habit and self-control, which are known barriers in the transition of attitude to behaviour (57). Also, the weak relationship between knowledge and behaviour highlights the complexity of translating awareness into action, as previously noted by Kelly and Fussell (58). While awareness is foundational, meaningful behavioural changes require supportive systems, addressing competing motivations and overcoming structural barriers (59), which the African environment has not provided.

Our study found a poor level of knowledge, a negative attitude, and an unsatisfactory behaviour towards air pollution reduction and its link to CVD among a large proportion of vulnerable populations of men and women stratified by occupation as a mode of exposure to air pollution. The results obtained may not reflect the situation among other populations that are less exposed to air pollution. Nevertheless, it is likely that the population studied would be more knowledgeable and exhibit a positive attitude and behaviour towards air pollution than the less exposed (60). Therefore, our study presents a unique opportunity for future longitudinal studies in the general population to develop culturally sensitive interventions to mitigate the cardiovascular health impact of exposure to air pollution.

## Strengths and Limitations

This study’s originality and contribution to existing knowledge are major strengths. There is limited research or evidence-based information on community individuals’ KAB towards the cardiovascular benefits of reduced exposure to air pollution in Africa; therefore, this study makes an original contribution to fill this evidence gap in this setting, as well as offering insight into other LMIC settings that may face similar issues. Also, the uniqueness and the sample size of the population studied offer invaluable insights into understanding the complex interplay between environmental awareness and individual actions by identifying the knowledge gap and developing tailored educational interventions. In addition, the AirHealth questionnaire’s extensive validation process, including face validity, content validity, and pilot testing, ensures that the data collected are reliable and valid.

A notable limitation of this study is the occupation-specific grouping that was used in the multi-stage sampling strategy. Although this method is practical, it may introduce selection bias because participants could have similar views or backgrounds, potentially resulting in a non-representative sample. However, efforts were made to mitigate potential bias through further stratification of the population to include a wide range of views and backgrounds. In addition, the absence of qualitative insight in the current study limits the interpretability of the findings, given that attitude and behaviour are mostly socio-culturally influenced. Future mixed-method studies are therefore advocated to provide a comprehensive overview of the cardiovascular benefits of reduced exposure to air pollution. Furthermore, we acknowledged the use of some double-barrelled questions to assess attitude and preventive behaviour, which might impact the specificity of the findings. However, its effect on the validity of the questions and reliability of the findings was mitigated by building the capacity of the RAs and the data team with the knowledge and skills to carefully interpret the questions and findings, in addition to organising a data interpretation meeting with the CARDINAL community advisory network (CAN) and community support groups (CSGs) to allow for data clarity and accuracy.

## Conclusion

Community members had poor knowledge, negative attitudes and unsatisfactory behaviour towards the cardiovascular benefits of reduced exposure to air pollution. However, awareness about air pollution was independently associated with good knowledge of the link between air pollution and CVD, while sex disparity was implicated in behaviour towards air pollution reduction. Therefore, strengthening community mobilisation and advocacy may improve their KAB. However, additional studies are warranted to determine the extent to which community engagement can improve KAB. Given the current situation on air pollution and its importance as a major public health concern, studies from Africa are required to identify culturally sensitive strategies to educate the general community to recognise the impact of environmental exposures on health outcomes.

## Implications and Recommendations

This study underscores the need for awareness campaigns, policy enforcement and gender-sensitive interventions in improving air pollution-related behaviours in Nigeria and potentially other African communities. Strengthening public health communication, enhancing access to clean energy sources and fostering community engagement will all be vital steps towards reducing air pollution and its associated cardiovascular risks. Our study provides a foundation for a larger upcoming study that will assess the impact of educational intervention. These findings underscore the multifaceted nature of the factors that influence KAB related to air pollution among vulnerable populations of men and women. Understanding these relationships is essential for developing effective targeted interventions and educational strategies to enhance environmental awareness and promote healthier behaviours among vulnerable populations. These insights reinforce the need for policies to support air quality measures, such as subsidising biogas access or integrating air pollution education into primary healthcare.

## Additional File

The additional file for this article can be found as follows:

10.5334/gh.1482.s1 Supplementary Files. Tables S1, S2 and Figure S1.

## References

[1] Brauer M, Roth GA, Aravkin AY, Zheng P, Abate KH, Abate YH, et al. Global burden and strength of evidence for 88 risk factors in 204 countries and 811 subnational locations, 1990–2021: A systematic analysis for the Global Burden of Disease Study 2021. Lancet. 2024;403(10440):2162–2203. DOI: 10.1016/S0140-6736(24)00933-4 38762324 PMC11120204

[2] (UN) UN. Department of Economic and Social Affairs, and Population Division, World Urbanization Prospects: The 2018 Revision. United Nations, New York; 2019.

[3] Sun B, Fang C, Liao X, Guo X, Liu Z. The relationship between urbanization and air pollution affected by intercity factor mobility: A case of the Yangtze River Delta region. Environ Impact Assess Rev. 2023;100:107092. DOI: 10.1016/j.eiar.2023.107092

[4] Dimitroulopoulou S, Dudzińska MR, Gunnarsen L, Hägerhed L, Maula H, Singh R, et al. Indoor air quality guidelines from across the world: An appraisal considering energy saving, health, productivity, and comfort. Environ Int. 2023;178:108127. DOI: 10.1016/j.envint.2023.108127 37544267

[5] World Health Organization. WHO global air quality guidelines: particulate matter (PM2.5 and PM10), ozone, nitrogen dioxide, sulfur dioxide and carbon monoxide. Geneva: WHO; 2021. 34662007

[6] Bereitschaft B, Debbage K. Urban form, air pollution, and CO2 emissions in large U.S. metropolitan areas. Prof Geogr. 2013;65(4):612–635. DOI: 10.1080/00330124.2013.799991

[7] (WHO) WHO. Ambient (outdoor) air pollution. [cited 2025 February 3]. Available from: https://www.who.int/news-room/fact-sheets/detail/ambient-(outdoor)-air-quality-and-health

[8] Liu Y, Ning N, Sun T, Guan H, Liu Z, Yang W, et al. Association between solid fuel use and nonfatal cardiovascular disease among middle-aged and older adults: Findings from The China Health and Retirement Longitudinal Study (CHARLS). Sci Total Environ. 2023;856(Pt 2):159035. DOI: 10.1016/j.scitotenv.2022.159035 36191716

[9] Balmes JR. Household air pollution from domestic combustion of solid fuels and health. J Allergy Clin Immunol. 2019;143(6):1979–1987. DOI: 10.1016/j.jaci.2019.04.016 31176380

[10] Bonjour S, Adair-Rohani H, Wolf J, Bruce Nigel G, Mehta S, Prüss-Ustün A, et al. Solid fuel use for household cooking: Country and regional estimates for 1980–2010. Environ Health Perspect. 2013;121(7):784–790. DOI: 10.1289/ehp.1205987 23674502 PMC3701999

[11] Naz L, Ghimire U. Assessing the prevalence trend of childhood pneumonia associated with indoor air pollution in Pakistan. Environ Sci Pollut Res Int. 2020;27(35):44540–44551. DOI: 10.1007/s11356-020-10346-6 32770471

[12] Lim S, Barratt B, Holliday L, Griffiths CJ, Mudway IS. Characterising professional drivers’ exposure to traffic-related air pollution: Evidence for reduction strategies from in-vehicle personal exposure monitoring. Environ Int. 2021;153:106532. DOI: 10.1016/j.envint.2021.106532 33812042

[13] Lawin H, Ayi Fanou L, Hinson AV, Stolbrink M, Houngbegnon P, Kedote NM, et al. Health risks associated with occupational exposure to ambient air pollution in commercial drivers: A systematic review. Int J Environ Res Public Health. 2018;15(9):2039. DOI: 10.3390/ijerph15092039 30231523 PMC6163743

[14] Schraufnagel DE, Balmes JR, Cowl CT, De Matteis S, Jung SH, Mortimer K, et al. Air pollution and noncommunicable diseases: A review by the forum of international respiratory societies’ environmental committee, Part 2: Air pollution and organ systems. Chest. 2019;155(2):417–426. DOI: 10.1016/j.chest.2018.10.041 30419237 PMC6904854

[15] Chen J, Atkinson RW, Andersen ZJ, Oftedal B, Stafoggia M, Lim Y-H, et al. Long-term exposure to ambient air pollution and risk of lung cancer – A comparative analysis of incidence and mortality in four administrative cohorts in the ELAPSE study. Environ Res. 2024;263(Pt 3):120236. DOI: 10.1016/j.envres.2024.120236 39455045

[16] Liu CS, Wei Y, Danesh Yazdi M, Qiu X, Castro E, Zhu Q, et al. Long-term association of air pollution and incidence of lung cancer among older Americans: A national study in the Medicare cohort. Environ Int. 2023;181:108266. DOI: 10.1016/j.envint.2023.108266 37847981 PMC10691920

[17] Fakunle AG, Olusola B, Jafta N, Faneye A, Heederik D, Smit LAM, et al. Home assessment of indoor microbiome (HAIM) in relation to lower respiratory tract infections among under-five children in Ibadan, Nigeria: The study protocol. Int J Environ Res Public Health. 2020;17(6):1857. DOI: 10.3390/ijerph17061857 32183028 PMC7143126

[18] Fakunle AG, Jafta N, Smit LAM, Naidoo RN. Indoor bacterial and fungal aerosols as predictors of lower respiratory tract infections among under-five children in Ibadan, Nigeria. BMC Pulm Med. 2022;22(1):471. DOI: 10.1186/s12890-022-02271-w 36494686 PMC9733100

[19] Adeoye AM FA, Aderonmu O, Tayo B. Short-term exposure to household air pollution and risk of hypertension among adults: A pilot study in Ibadan. J Health Environ Res. 2020;6(2):37–43. DOI: 10.11648/j.jher.20200602.12

[20] Alexeeff SE, Deosaransingh K, Van Den Eeden S, Schwartz J, Liao NS, Sidney S. Association of long-term exposure to particulate air pollution with cardiovascular events in California. JAMA Netw Open. 2023;6(2):e230561. DOI: 10.1001/jamanetworkopen.2023.0561 36826819 PMC9958530

[21] Şahin B, İlgün G. Risk factors of deaths related to cardiovascular diseases in World Health Organization (WHO) member countries. Health Soc Care Community. 2022;30(1):73–80. DOI: 10.1111/hsc.13156 32909378

[22] Bourdrel T, Bind MA, Béjot Y, Morel O, Argacha JF. Cardiovascular effects of air pollution. Arch Cardiovasc Dis. 2017;110(11):634–642. DOI: 10.1016/j.acvd.2017.05.003 28735838 PMC5963518

[23] Brook RD, Franklin B, Cascio W, Hong Y, Howard G, Lipsett M, et al. Air pollution and cardiovascular disease: A statement for healthcare professionals from the Expert Panel on Population and Prevention Science of the American Heart Association. Circulation. 2004;109(21):2655–2671. DOI: 10.1161/01.CIR.0000128587.30041.C8 15173049

[24] Lala MA, Onwunzo CS, Adesina OA, Sonibare JA. Particulate matters pollution in selected areas of Nigeria: Spatial analysis and risk assessment. Case Stud Chem Environ Eng. 2023;7:100288. DOI: 10.1016/j.cscee.2022.100288

[25] Onakpohor A, Fakinle BS, Sonibare JA, Oke MA, Akeredolu FA. Investigation of air emissions from artisanal petroleum refineries in the Niger-Delta Nigeria. Heliyon. 2020;6(11):e05608. DOI: 10.1016/j.heliyon.2020.e05608 33299937 PMC7702015

[26] Giwa SO, Nwaokocha CN, Kuye SI, Adama KO. Gas flaring attendant impacts of criteria and particulate pollutants: A case of Niger Delta region of Nigeria. J King Saud Univ Eng Sci. 2019;31(3):209–217. DOI: 10.1016/j.jksues.2017.04.003

[27] NBS. Road industry data. Nigeria Bureau Statistics; (2019). [cited 2025 February 3]. Available from: https://wwwnigerianstatgovng/pdfuploads/Road_Transport_Data_Q4_2018pdf

[28] Croitoru L, Chang JC, Kelly A, editors. The cost of air pollution in Lagos 2020. DOI: 10.1596/33038

[29] Fabrigar L, Petty R, Smith S, Crites S. Understanding knowledge effects on attitude-behavior consistency: The role of relevance, complexity, and amount of knowledge. J Pers Soc Psychol. 2006;90(4):556–577. DOI: 10.1037/0022-3514.90.4.556 16649855

[30] Fakunle AG, Bello T, Ogunwale AO, Morakinyo O, Ayinde O, Kebu S, et al. Community-based cardiovascular risk reduction through novel air health behavioural change intervention (CARDINAL): Study protocol. Ann Med Health Sci Res. 2024;14:981–989.

[31] Oltra C, Sala R. Perception of risk from air pollution and reported behaviors: A cross-sectional survey study in four cities. J Risk Res. 2016;21(7):1–16. DOI: 10.1080/13669877.2016.1264446

[32] Gatersleben B, Uzzell D. The risk perception of transport–generated air pollution. IATSS Res. 2000;24(1):30–38. DOI: 10.1016/S0386-1112(14)60015-7

[33] Al-Shidi HK, Ambusaidi AK, Sulaiman H. Public awareness, perceptions and attitudes on air pollution and its health effects in Muscat, Oman. J Air Waste Manag Assoc. 2021;71(9):1159–174. DOI: 10.1080/10962247.2021.1930287 33989134

[34] Lynch KM, Mirabelli MC. Outdoor air quality awareness, perceptions, and behaviors among U.S. children aged 12–17 years, 2015–2018. J Adolesc Health. 2021;68(5):882–887. DOI: 10.1016/j.jadohealth.2020.07.040 32919887 PMC7940452

[35] Shabani Isenaj Z, Moshammer H, Berisha M, Weitensfelder L. Determinants of knowledge, attitudes, perceptions and behaviors regarding air pollution in schoolchildren in Pristina, Kosovo. Children (Basel, Switzerland). 2024.11(1):128. DOI: 10.3390/children11010128 38275438 PMC10814697

[36] Rendon-Marin S, Higuita-Gutiérrez LF, Gomez-Gallego DM. Knowledge, attitudes, and practices regarding air pollution among medical students. Int J Environ Res Public Health. 2024;21(6):789. DOI: 10.3390/ijerph21060789 38929035 PMC11204335

[37] Susan Paul NS, Mathew P, Johns F, Abraham J. The feasibility of using remote data collection tools in field surveys. Int J Commun Med Public Health. 2017;5(1):81–85. DOI: 10.18203/2394-6040.ijcmph20175514

[38] Nassirou-Sabo H, Toudou-Daouda M. Assessment of knowledge, attitudes, and practices of occupational risks and diseases among healthcare providers of the Regional Hospital Center of Dosso, Niger. SAGE Open Medicine. 2024;12:20503121231224549. DOI: 10.1177/20503121231224549 38288477 PMC10823837

[39] Debsarma D. Exploring the strategies for upgrading the rural unqualified health practitioners in West Bengal, India: A knowledge, attitude and practices assessment-based approach. Health Policy Open. 2022;3:100083. DOI: 10.1016/j.hpopen.2022.100083 37383573 PMC10297518

[40] Kaur TP, Rana A, Perumal V, Sharma A, Dadhwal V, Kulshrestha V, et al. A cross-sectional analysis to evaluate knowledge, attitude and practices among pregnant women during COVID-19 pandemic. J Obstet Gynaecol India. 2021;71(Suppl 1):18–27. DOI: 10.1007/s13224-021-01558-y 34511781 PMC8416569

[41] Adeoye M, Rahimzadeh S, Taylor S, Shrikhande S, Perel P, Shah A, et al. The impact of air pollution on cardiovascular health outcomes in African populations: A scoping review. JACC Adv. 2024;3(12, Part 2):101371. DOI: 10.1016/j.jacadv.2024.101371 39817083 PMC11733974

[42] Manisalidis I, Stavropoulou E, Stavropoulos A, Bezirtzoglou E. Environmental and health impacts of air pollution: A review. Front Public Health. 2020;8:14. DOI: 10.3389/fpubh.2020.00014 32154200 PMC7044178

[43] Farag D, Akpede N, Waterson H, Asogun D, Faustina Funmilayo B, Nnadi C, et al. The impact of education level on knowledge, attitudes and practices towards COVID-19 in Edo State, Nigeria. J Public Health (Oxford, England). 2023;45(Suppl 1):i63–i70. DOI: 10.1093/pubmed/fdac142 38127562

[44] Kim JB, Prunicki M, Haddad F, Dant C, Sampath V, Patel R, et al. Cumulative lifetime burden of cardiovascular disease from early exposure to air pollution. J Am Heart Assoc. 2020;9(6):e014944. DOI: 10.1161/JAHA.119.014944 32174249 PMC7335506

[45] Ramírez AS, Ramondt S, Van Bogart K, Perez-Zuniga R. Public awareness of air pollution and health threats: Challenges and opportunities for communication strategies to improve environmental health literacy. J Health Commun. 2019;24(1):75–83. DOI: 10.1080/10810730.2019.1574320 30730281 PMC6688599

[46] Agbo KE, Walgraeve C, Eze JI, Ugwoke PE, Ukoha PO, Van Langenhove H. A review on ambient and indoor air pollution status in Africa. Atmos Pollut Res. 2021;12(2):243–260. DOI: 10.1016/j.apr.2020.11.006

[47] Marín D, Calle N, Arango V, Betancur P, Pérez M, Orozco LY, et al. Knowledge, attitudes and practices about air pollution and its health effects in 6th to 11th-grade students in Colombia: A cross-sectional study. Front Public Health. 2024;12:1390780. DOI: 10.3389/fpubh.2024.1390780 38962783 PMC11221384

[48] Alves RF. The relationship between health-related knowledge and attitudes and health risk behaviours among Portuguese university students. Global Health Promot. 2023;31(1):36–44. DOI: 10.1177/17579759231195561 PMC11015703 37715622

[49] Ayanaw Eyayu R, Gudayu Zeleke T, Chekol WB, Yaregal Melesse D, Enyew Ashagrie H. Assessment of level of knowledge, attitude, and associated factors toward delirium among health professionals working in intensive care unit multicenter, cross-sectional study, Amhara region comprehensive specialized hospitals, Northwest Ethiopia, 2023. Front Public Health. 2024;12:1338760. DOI: 10.3389/fpubh.2024.1338760 38510361 PMC10951067

[50] Marcantonio R, Javeline D, Field S, Fuentes A. Global distribution and coincidence of pollution, climate impacts, and health risk in the Anthropocene. PLoS One. 2021;16(7):e0254060. DOI: 10.1371/journal.pone.0254060 34288922 PMC8294505

[51] Vlaev I, King D, Darzi A, Dolan P. Changing health behaviors using financial incentives: A review from behavioral economics. BMC Public Health. 2019;19(1):1059. DOI: 10.1186/s12889-019-7407-8 31391010 PMC6686221

[52] Endalew M, Belay DG, Tsega NT, Aragaw FM, Gashaw M, Asratie MH. Household Solid Fuel Use and Associated Factors in Ethiopia: A Multilevel Analysis of Data From 2016 Ethiopian Demographic and Health Survey. Environ Health Insights. 2022;16:11786302221095033. DOI: 10.1177/11786302221095033 35521361 PMC9067044

[53] Wu S. The health impact of household cooking fuel choice on women: Evidence from China. Sustainability. 2021;13(21). DOI: 10.3390/su132112080

[54] Yu C, Long H, Zhang X, Tan Y, Zhou Y, Zang C, et al. The interaction effect between public environmental concern and air pollution: Evidence from China. J Clean Prod. 2023;391:136231. DOI: 10.1016/j.jclepro.2023.136231

[55] Isezuo S, Sani MU, Talle A, Johnson A, Adeoye AM, Ulgen MS, et al. Registry for Acute Coronary Events in Nigeria (RACE-Nigeria): Clinical characterization, management, and outcome. J Am Heart Assoc. 2022;11(1):e020244. DOI: 10.1161/JAHA.120.020244 34935419 PMC9075212

[56] Global burden of 87 risk factors in 204 countries and territories, 1990–2019: A systematic analysis for the Global Burden of Disease Study 2019. Lancet (London, England). 2020;396(10258):1223–1249. DOI: 10.1016/S0140-6736(20)30752-2 33069327 PMC7566194

[57] Itzchakov G, Uziel L, Wood W. When attitudes and habits don’t correspond: Self-control depletion increases persuasion but not behavior. J Exp Soc Psychol. 2018;75:1–10. DOI: 10.1016/j.jesp.2017.10.011

[58] Kelly FJ, Fussell JC. Air pollution and public health: Emerging hazards and improved understanding of risk. Environ Geochem Health. 2015;37(4):631–649. DOI: 10.1007/s10653-015-9720-1 26040976 PMC4516868

[59] Happell B, Stanton R, Hoey W, Scott D. Knowing is not doing: The relationship between health behaviour knowledge and actual health behaviours in people with serious mental illness. Mental Health and Physical Activity. 2014;7(3):198–204. DOI: 10.1016/j.mhpa.2014.03.001

[60] Hashem RB, Siddique AB, Rasel SM, Hossain MS. Assessment of knowledge, attitudes, and practices regarding air pollution among traffic polices in Dhaka city, Bangladesh: A cross-sectional study. BMC Public Health. 2024;24(1):3593. DOI: 10.1186/s12889-024-21086-4 39731081 PMC11673892

