# Supplementary material for: Community Knowledge, Attitudes and Preventive Behaviour Towards the Cardiovascular Benefits of Reduced Exposure to Air Pollution in Nigeria: Evidence from the CARDINAL Study
Source: Glob Heart. 2025 Oct 9;20(1):90. doi: 10.5334/gh.1482 (PMC12513364; doi:10.5334/gh.1482)
Supplement: Supplementary Files. — Tables S1, S2 and Figure S1. [file gh-20-1-1482-s1.pdf]

Community knowledge, attitudes and preventive behaviour towards the cardiovascular benefits of reduced exposure to air pollution in Nigeria: evidence from the CARDINAL study

Supplementary Materials

Table S1: Bivariate analysis of the socio-demographic characteristics associated with KAB categories

Socio-demographic characteristics	Knowledge		*P*	Attitude		*P*	Behaviour		*P*
	Poor; n (%)	Good; n (%)		Negative; n (%)	Positive; n (%)		Unsatisfactory; n (%)	Satisfactory; n (%)	
**Age (years)**									
≤50	264 (65.0)	113 (56.2)	**0.035**	224 (63.1)	153 (60.7)	0.551	210 (59.0)	167 (66.5)	0.059
>50	142 (35.0)	88 (43.8)		131 (36.9)	99 (39.9)		146 (41.0)	84 (33.5)	
**Mean ± SD**	44.08 ± 17.03	47.64 ± 16.88	**0.016**	44.87 ± 17.18	45.81 ± 16.88	0.507	46.49 ± 17.63	43.51 ± 16.06	0.065
**Sex**									
Male	189 (46.6)	89 (44.3)	0.599	224 (63.1)	153 (60.7)	0.551	143 (40.2)	135 (53.8)	**0.001**
Female	217 (53.4)	112 (55.7)		131 (36.9)	99 (39.3)		213 (59.8)	116 (46.2)	
**Religion**									
Christianity	137 (33.7)	50 (24.9)	**0.033**	166 (32.0)	21 (23.9)	0.057	122 (31.8)	65 (29.3)	0.590
Islam	269 (66.3)	150 (74.6)		353 (68.0)	66 (75.0)		261 (68.0)	157 (70.7)	
Traditional	0 (0.0)	1 (0.5)		0 (0.0)	1 (1.1)		1 (0.3)	0 (0.0)	
**Ethnicity**									
Yoruba	359 (88.4)	173 (86.1)	**0.013**	386 (86.2)	226 (89.7)	**0.021**	311 (87.1)	221 (88.0)	0.968
Igbo	47 (11.6)	28 (13.4)		14 (3.9)	1 (0.4)		9 (2.1)	6 (2.4)	
Hausa	33 (8.1)	27 (13.4)		35 (9.9)	25 (9.9)		36 (10.1)	24 (9.6)	
**Type of Family**									
Monogamy	313 (77.1)	134 (66.7)	**0.006**	273 (76.9)	174 (69.0)	**0.030**	256 (71.9)	191 (76.1)	0.249
Polygamy	93 (22.9)	67 (33.3)		82 (23.1)	78 (31.0)		100 (28.1)	60 (23.9)	
**Highest Level of Education**									
No Formal Education	52 (12.8)	34 (16.9)	**0.001**	49 (13.8)	37 (14.7)	0.974	62 (17.4)	24 (9.6)	**<0.001**
Primary	90 (22.2)	68 (33.8)		91 (25.6)	67 (26.6)		104 (29.2)	54 (21.5)	

Secondary	214 (52.7)	87 (43.3)		178 (50.1)	123 (48.8)		163 (45.8)	138 (55.0)	
Tertiary	50 (12.3)	12 (6.0)		37 (10.4)	25 (9.9)		27 (7.6)	35 (13.9)	
**Air pollution awareness**									
Yes	252 (62.1)	126 (62.7)	**0.004**	222 (62.5)	156 (61.9)	0.875	192 (53.9)	186 (74.1)	**<0.001**
No	154 (37.9)	75 (37.3)		133 (37.5)	96 (38.1)		164 (46.1)	65 (25.9)	
**Household Size**									
< 5	254 (62.9)	131 (65.2)	0.579	213 (60.2)	172 (68.5)	**0.035**	232 (65.4)	153 (61.2)	0.296
≥ 5	150 (37.1)	70 (34.8)		141 (39.8)	79 (31.5)		123 (34.6)	97 (38.8)	
**Respondent's monthly income ($)**									
<100	369 (91.1)	190 (94.5)	0.139	331 (93.2)	228 (90.8)	0.276	325 (91.5)	234 (93.2)	0.447
≥100	36 (8.9)	11 (5.5)		24 (6.8)	23 (9.2)		30 (8.5)	17 (6.8)	
**Length of stay in year**									
< 1	28 (6.9)	21 (10.4)	0.056	12 (2.3)	5 (5.7)	0.158	10 (2.6)	7 (3.2)	0.630
1-3	49 (12.1)	19 (9.5)		90 (17.3)	10 (11.4)		58 (15.1)	42 (18.9)	
4-6	62 (15.3)	18 (9.0)		70 (13.5)	10 (11.4)		52 (13.5)	28 (12.6)	
>= 7	267 (65.8)	143 (71.1)		347 (66.9)	63 (71.6)		264 (68.8)	145 (65.3)	

Table S2: Correlation analysis

	**Knowledge**	**Attitude**	**Behaviour**
**Knowledge**	1.00		
**Attitude**	0.32<br>**<0.001**	1.00	
**Behaviour**	0.13<br>**0.001**	0.04<br>0.33	1.00

Spearman's correlation analysis showing the correlation coefficient (rho) and the p-value

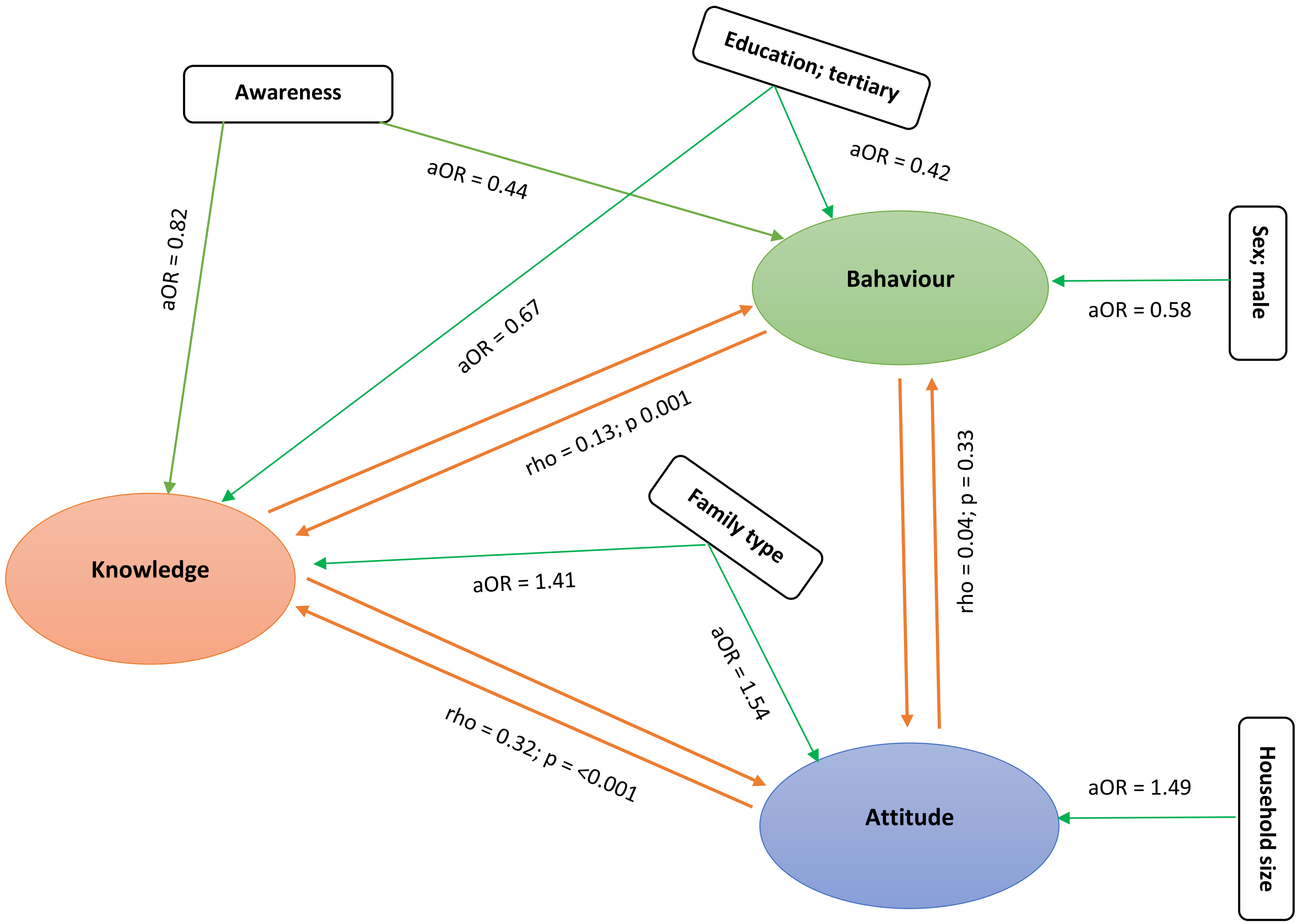

Figure S1: 1) red lines representing correlation between knowledge, attitude and behaviour with the correlation coefficient and p-value; and 2) green lines representing socio-demographic and socioeconomic factors associated with knowledge, attitude and behaviour in a multivariable-adjusted regression analysis. For all variables, the p-value was <0.05.
